# Prevention of mental health issues in the young: A randomised controlled evaluation of an e-mental health application for young adults to enhance mental health literacy

**DOI:** 10.1016/j.invent.2025.100874

**Published:** 2025-09-17

**Authors:** Olivia Krokos, Isabel Brandhorst, Caterina Gawrilow, Johanna Löchner

**Affiliations:** a Department of School Psychology, Tübingen, Germany; b Department of Child and Adolescent Psychiatry, Psychosomatics and Psychotherapy, University Hospital, Tübingen, Germany; c Friedrich-Alexander University, Erlangen-Nuremberg, Germany; d German Center for Mental Health (DZPG), partner site Tübingen, Germany

**Keywords:** mHealth, Mental health literacy, Young adulthood, Prevention

## Abstract

**Background:**

The mental health of young adults is deteriorating. Reasons for this are manifold, ranging from biological factors (e.g. entering a vulnerable developmental phase) to crisis-related external events (e.g. COVID-19 pandemic). Accordingly, easily accessible and universal prevention for the young is needed. Mobile Health (mHealth) interventions are on the rise and especially promising for this age group, due to numerous benefits, such as low threshold, temporal and local flexibility and high scalability. However, the effectiveness and acceptance of mHealth interventions as prevention measures are missing empirical evidence.

**Method:**

In a two-arm randomised controlled trial design, this study aimed to evaluate the effectiveness of a mental health app, the ‘Mental Health Guide’, primarily on mental health literacy as well as secondary mental health outcomes. *N* = 322 Participants (81.99 % female, M = 25.55 years, SD = 9.63 years, age range: 15 to 59 years) were either assigned to the intervention group (*n* = 158), using the Mental Health Guide for 12 weeks, or the wait-list control group (*n* = 164).

**Results:**

The results show a significant intervention effect on mental health literacy for the intervention group in the post assessment (*p* = .047, *d* = 0.20), but no at later follow-up time points. Further variables related to mental health indicate various effects, such as improved problematic (*p* = .018, *d* = 0.20) and prosocial behaviour (*p* = .008, *d* = 0.23) in the intervention group and improved emotion regulation capacities for both groups (*p* < .001, *d* = 0.20). Overall, there was a high drop-out rate in the study (up to 80 %), especially in the intervention group.

**Conclusion:**

This study contributes valuable insights into the potential effectiveness of mHealth prevention in young adults and gives insights on how such applications are used under very naturalistic settings, laying a foundation for future research in this field. However, generalisability is limited due to selective sample characteristics and a rather high drop-out rate over time.

## Highlights

• The use of mobile health measures showed short-term effectiveness on mental health literacy.
• Mobile health app use reduced problematic behaviour and increased prosocial behaviour over time.
• Higher app acceptance was linked to better well-being over time.
• EMA provided valuable real-time insights into mood and emotion regulation dynamics.

## 1 Introduction

Mental health and overall well-being of young adults are in decline. Since the onset of the COVID-19 pandemic, the mental health status of this age group in Germany has deteriorated to levels comparable to those last observed in 2010 (Beckmannshagen and Graeber, 2024). At the same time, the age of onset for most mental disorders peaks at 14.5 years on average (Solmi et al., 2022). To counteract this downward trend, interventions such as prevention strategies targeting young adults are needed to strengthen mental health from an early stage.

Good well-being is crucial for the vulnerable developmental phase from adolescence to adulthood (Fuchs and Karwautz, 2017). However, maintaining good mental health is challenged by various risk factors, the most common being stress, anxiety and difficulties in emotion regulation, which influence the development of mental disorders (Patel et al., 2007). A fundamental way to address these challenges is by educating people about risk and protective factors that are linked to mental health and the development of mental disorders (Fuchs and Karwautz, 2017). Mental health literacy (MHL) is defined as the knowledge and beliefs individuals hold about mental health problems, including knowledge about risk and protective factors. Further related aspects of MHL are the ability to recognise specific disorders, knowledge of mental health information sources, knowledge of self-help strategies, as well as knowledge of available professional help and attitudes that promote recognition and help-seeking behaviours (Jorm, 2012). Good MHL enhances the search for and use of preventive services (Jorm et al., 1997), whereas the lack of knowledge, prejudice and stigmatisation of mental disorders lead to avoidance of psychotherapeutic services (Henderson et al., 2013). Among British university students, higher MHL leads to more help-seeking behaviour (Gorczynski et al., 2017) as well as more positive intentions and attitudes (Özparlak et al., 2023). Especially for help-seeking, considering depression and anxiety, MHL appears to be a significant facilitator (Lui et al., 2024). MHL not only improves access to mental health services but is also directly associated with higher mental well-being in adolescents (Whittaker et al., 2017).

As the strengthening of MHL should be considered a general educational aim, intervention strategies should target the universal population of young adults. Hence, universal prevention appears to be a reasonable measure for these purposes. In contrast to selective prevention (targeting subgroups with high-risk factors) and indicated prevention (targeting subgroups with first symptoms of mental illnesses), universal prevention targets the entire population (Siepmann, 2012). Meta-analyses indicate that universal prevention strategies can effectively reduce the incidence of mental disorders with small, but significant effect sizes in post-assessments, e.g. for individual or group face-to-face interventions in school settings (Ise et al., 2012; Hetrick et al., 2016). These interventions can also enhance understanding of mental disorders (Schiller et al., 2014). In addition, various prevention studies show positive effects on the onset and symptoms of mental disorders, e.g. by halving the incidence of depression in the treatment group compared to the control group in the follow-up (Weisz et al., 2005; Brent and Melhem, 2023). Considering various characteristics of quality of life, universal prevention can promote a more health-conscious lifestyle as well as the improvement and maintenance of occupational performance (Jorm, 2012). Therefore, universal prevention offers a low-cost, scalable and applicable approach, although effect sizes and long-term effects are lower than those found in selective and indicated prevention (Hetrick et al., 2016; Loechner et al., 2018).

To potentially achieve universal prevention for the whole population, the use of digital technology appears to be a promising option: Around 93 % of adolescents and young adults between the ages of 12 to 19 own a smartphone (Medienpädagogischer Forschungsverbund Südwest, 2024). The so-called mobile health (mHealth) interventions gained increasing interest since the COVID-19 pandemic, with over 40,000 different mHealth Apps currently being offered in the App Store and the Google Play Store (Mikulic, 2022). More than 69 % of 14- to 22-year-olds report using mHealth apps (Rideout et al., 2021; Wang et al., 2023). The advantages of mHealth interventions complement the aims of universal prevention with low-threshold access, geographically independent availability (Paganini et al., 2018), as well as temporal and locational flexibility and the promotion of self-determination (Robinson et al., 2016). Although mHealth interventions offer numerous advantages and app downloads and usage have continued to increase, empirical evidence on the feasibility and effectiveness of mHealth interventions remains limited (Larsen et al., 2019). The few existing studies mainly focus on the effectiveness of mHealth prevention targeting specific mental disorders, e.g. depression and anxiety disorder (Torous et al., 2020) or alcohol and substance abuse (Kazemi et al., 2017), rather than mental health in general. A meta-analysis including ten randomised controlled trials in the general (adult) population revealed similar effects as face-to-face interventions with small but positive effects for post-assessment for anxiety and depression outcomes (Deady et al., 2017). In contrast, universal effects did not differ significantly from indicated/selective prevention programs. As research on children and adolescents has often lagged behind, to our knowledge, there is only one quantitative study in Europe on the effectiveness of mHealth interventions for universal prevention purposes (Watkins et al., 2024a), as well as one universal prevention study in New Zealand, targeting the onset of depression in adolescents (Whittaker et al., 2017). Another mHealth approach focused on the improvement of emotional competencies with a self-help app but did not find significant changes in mental well-being (Watkins et al., 2024b). As prevention is mandatory in Australian schools, several large-scale trials have been conducted, such as the Health4Life intervention, which adopts a holistic approach to health encompassing nutrition, physical activity, and psychological strategies (Teesson et al., 2020). From a qualitative perspective, mHealth interventions generally seem to be an appropriate approach and an additional resource in prevention efforts (Beames et al., 2023). Challenging in the evaluation of mHealth interventions for universal prevention strategies is the high drop-out rate and low adherence (Moshe et al., 2021; Pauley et al., 2023). In order to increase adherence as much as possible and to keep drop-out low, further app characteristics, besides quality of information and acceptance of intervention, such as app design, delivery method and guidance, need to be investigated (Grist et al., 2017).

This study aimed to evaluate the effectiveness of an mHealth intervention as a universal prevention method for young adults. Specifically, this randomised controlled trial aimed to assess whether the preventive use of a mHealth app can increase MHL and thereby promote psychological well-being. One such mHealth intervention is the app *Mental Health Guide* (for further information, see also https://www.mentalhealthcrowd.de/was-wir-tun/mentalhealthguide/). The app is intended as a universal digital prevention tool to strengthen mental health and improve MHL. Although the app offers to be attractive and suitable for the target group of young adults, scientific studies that test both usability and effectiveness of the app are still pending. Primarily, we hypothesised that the use of the app leads to an increased MHL in young adults aged 14 years and older. Secondly, we hypothesised that usage of the app would have a positive impact on mental health, including improved emotion regulation skills, reduced psychological distress, and improved psychological well-being. In addition, the usability and acceptance of the app as an mHealth product were investigated exploratorily.

## 2 Methods

### 2.1 Study design and consent

**Fig. 1:**
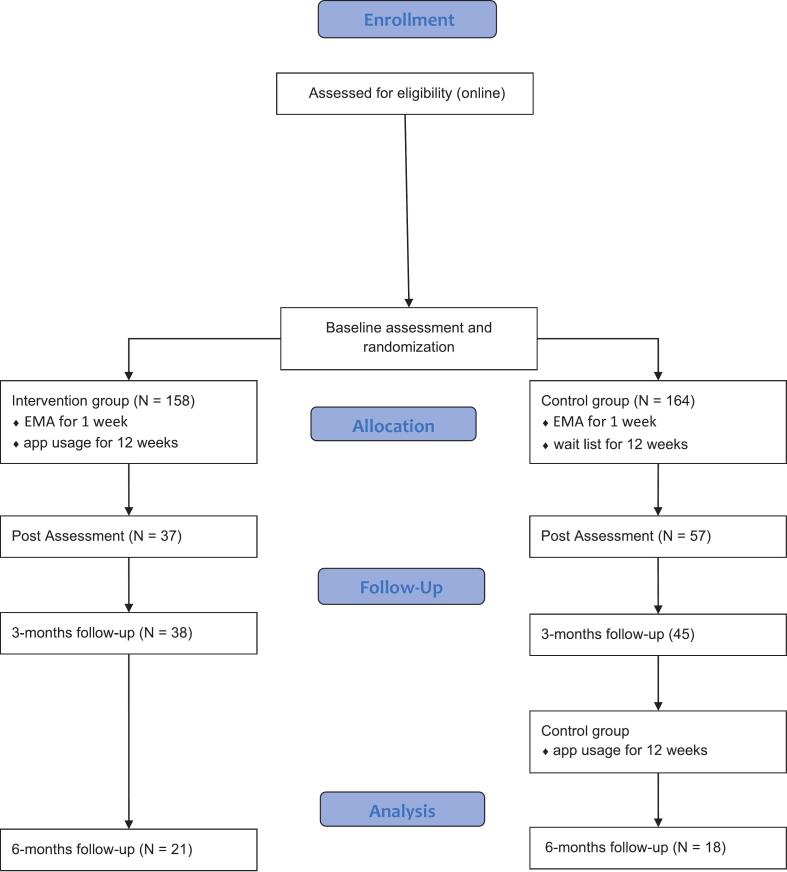
Flowchart of the study design.

The study was performed as a randomised controlled trial to promote MHL and strengthen the mental health of young adults, as well as to evaluate the usability and acceptance of the app. The trial has a parallel two-arm design (see Fig. 1), with participants being randomly assigned by a computerised random generator to either the intervention group (IG) or the waiting list control group (CG). To avoid measurement biases (e.g., memory bias) but to capture more information on dynamic behaviour changes (Seizer et al., 2024), ecological momentary assessment (EMA) was implemented. The study was carried out in line with the Declaration of Helsinki (World Medical Association Declaration of Helsinki, 2013) and has received approval from the ethical committee of the Universitätsklinikum Tübingen (Ref no. 842/2022BO1, June 2023), as well as from the German Ministry of education (Ref. no. KM31-6499-3/115/3). The study was registered in the German register for clinical trials (DRKS-ID: DRKS00031810).

### 2.2 Recruitment and sample

Participants were recruited via high schools and a university in the south of Germany (Baden-Württemberg), as well as social media channels. To participate in the study, interested participants had to be at least 14 years old, have an internet-enabled device, and have sufficient German language skills according to their own subjective assessment. Before participating, all participants received information sheets, agreed to the study data protection conditions and provided written informed consent. Participants under the age of 18 years also provided parental consent. In total, 322 participants (264 female, 52 male, and 3 diverse) took part in the study. Fig. 1 provides an overview of the respective sample sizes at each assessment point. Participants were on average 25.55 years old (SD = 9.63), with an age span from 15 to 59 years. Further demographic characteristics can be found in Table 1. As reimbursement, all participants continued to receive unlimited free access to the *Mental Health Guide* after study completion.

**Table 1:** demographic characteristics.

	IG		CG	
	*n*	%	*n*	%
Gender				
Female	132	83.54	132	80.49
Male	23	14.56	29	17.68
Diverse	3	1.90	3	1.83
Occupation				
School	41	25.95	42	25.61
University	68	43.04	77	46.95
Employed	48	30.38	44	26.83
Other	1	0.63	1	0.61
Cultural affiliation				
German	150	94.94	153	93.29
European		1.27	1	0.61
Arabic	2	0.00	5	3.05
Asian	0	0.63	0	0.00
African	1			
Other	1	0.63	1	0.61
Not specified	4	2.53	4	2.44
Dealing with mental health	134	84.81	140	85.37
Experience with therapeutic services	77	48.73	81	49.39

Note. *N* = 322 with *n* = 158 in the Intervention group (IG) and *n* = 164 in the wait list control group (CG).

### 2.3 Procedure

The study took place from May 2023 until November 2024. After consenting to the study, participants received access to the online baseline questionnaires (T0). After completion, participants were randomly assigned by a computerised random generator, based on codes only, to either the IG or CG. For further insights into the dynamics of participants' general well-being, participants were prompted the following week for the EMA survey assessing participants' momentary well-being and emotion regulation capacities, predefined in a time-based sampling design (Seizer et al., 2024), each morning (7 am) and evening (9 pm). With the end of the EMA survey, participants received information about their group assignment, with the IG being told to download and use the *Mental Health Guide* at their discretion. The post assessment (T1) took place after three months. After that, both groups received further follow-up assessments 3 (T2) and 6 months (T3) after the post-assessment. The CG received access to the app after the post- and 3-month follow-up assessments. More detailed information about the study procedure and design can be found in the corresponding study protocol (Krokos et al., 2024).

### 2.4 Intervention

The *Mental Health Guide* is developed and distributed by a social company called the Mental Health Crowd with the help of experts from different domains (e.g., psychologists, coaches, therapists, experts with lived experience of mental illness) as a self-help e-learning course. The *Mental Health Guide* is usable as a progressive web application on a smartphone or as a desktop version via an internet browser. Containing 12 modules with different subsections (see Table 2), participants can learn which factors interact with their mental health and how they can influence it. The processing time of a single module varies between 20 and 60 min. The content of each module is presented in various text, image, and video formats, as well as quizzes and worksheets. There was no predefined order in which participants had to work through the modules. It was recommended to process one module per week, but participants decided for themselves when and how often they used the app. The study team that conducted the randomised controlled trial was and is not involved in the development or commercialisation of the *Mental Health Guide* and is independent of the Mental Health Crowd. In accordance with data protection regulations applicable in Germany, the study team had no access to individual-level usage data collected by the Mental Health Crowd. Consequently, the usage time of the app, as well as other objective data on usage, was not controlled by the study team.

**Table 2:** Summary of the 12 app modules.

Module	Content
#1 – Body	Healthy nutrition, exercise, sleep, breathing, stimulants
#2 – Stress and resources	(Physical) effects of stress, handling stress
#3 – Here and now	Mindfulness, change in life
#4 – Circumstances	Finances and consumer goods, geographical and temporal circumstances, human, mindset
#5 – Emotional intelligence	Fundamental information on emotions, emotions in myself and others
#6 – Needs	Fundamental information of human needs, communication of needs
#7 – Relationships with others	Roles in everyday life, social skills, forgiving
#8 – Relationship with me	Self-care, beliefs
#9 – Crisis	Dealing with crises, exercise for coping strategies
#10 – Psychoeducation	Information on symptoms and diagnostics of mental disorders, therapies and other treatment options
#11 – Communication	Fundamental information on communication and active listening, how to communicate about mental health
#12 – Values, goals, purpose	What are values, how to achieve goals, linking between values, goals and purposes

### 2.5 Outcome measures

Table 3 provides a list of all outcome measures.

**Table 3:** Summary of outcome measures.

Questionnaire	Baseline	EMA	Post	3 MFU	6 MFU
MHLS	X		X	X	X
SDQ	X		X	X	X
WHO-5	X		X	X	X
MDBF		X			
DERS-SF	X	X	X	X	X
RRS Brooding	X		X	X	X
PSS-4	X		X	X	X
MARS-G			X	X	X

Note. 3/6 MFU: 3/6 months follow-up*,* EMA: Ecological Momentary Assessment, MHLS: Mental Health Literacy Scale, MARS-G: Mobile App Rating Scale, SDQ: Strengths and Difficulties Questionnaire, DERS-SF: Difficulties in Emotion Regulation Scale -short form, RRS Brooding: Ruminative Response Scale Brooding, MDBF: Multidimensional Mood State Questionnaire, PSS-4: Perceived Stress Scale.

#### 2.5.1 Mental health literacy

The primary outcome was the 36-item Mental Health Literacy Scale (MHLS) divided into five dimensions of MHL, which are assessed using different Likert-Scales (O'Connor and Casey, 2015). A four-point Likert scale ranging from ‘1 = very unlikely/unhelpful’ to ‘4 = very likely/unhelpful’ is used for the dimensions ‘recognition of disorders’ (8 items), ‘knowledge of professional help available’ (3 items) and ‘knowledge of risk factors and causes’ (2 items). Five-point Likert scales ranging from ‘1 = strongly disagree/definitely unwilling’ to ‘5 = strongly agree/definitely willing’ are used for the dimensions ‘knowledge of how to seek mental health information’ (4 items) and ‘attitudes to promote recognition and appropriate help-seeking’ (16 items). The summary score ranges from 35 to 160, with higher scores indicating higher MHL. The original MHLS demonstrates good psychometric properties with an internal consistency of Cronbach's *α* = 0.873 (O'Connor and Casey, 2015). For this study, the German translation was retrieved from a master's thesis.

#### 2.5.2 Problematic and prosocial behaviour

As a secondary outcome, the German version of the Strengths and Difficulties Questionnaire (SDQ) assesses five behavioural dimensions (emotional symptoms, conduct problems, hyperactivity/impulsivity, peer relationship problems and prosocial behaviour) (Rothenberger et al., 2008). The SDQ consists of 25 items, assessed on a three-point Likert scale ranging from ‘0 = not true’ to ‘2 = certainly true’. The summary score ranges from 0 to 40, with scores between 0 and 15 indicating normal difficulties, 16 and 19 indicating borderline difficulties, and 20 and 40 indicating abnormal difficulties. The dimension ‘prosocial behaviour’ has an individual summary score between 0 and 10, with scores between 6 and 10 indicating normal prosocial behaviour, a score of 5 indicating borderline prosocial behaviour and a score between 0 and 4 indicating abnormal prosocial behaviour. The SDQ demonstrates acceptable psychometric properties with an internal consistency of Cronbach's *α* = 0.558 to 0.790 (Rothenberger et al., 2008).

#### 2.5.3 Overall well-being

A further secondary outcome was the German version of the WHO-5 Well-Being Index (WHO-5). It assesses overall well-being with five items on a six-point Likert scale ranging from ‘0 = all of the time’ to ‘5 = at no time’ (Brähler et al., 2007). The summary score ranges from 0 to 25, with higher scores indicating overall higher well-being. The WHO-5 demonstrates good psychometric properties with an internal consistency of Cronbach's *α* = 0.920 (Brähler et al., 2007).

#### 2.5.4 Current well-being

For EMA, the German Multidimensional Mood State Questionnaire (MDBF) assesses current well-being in its short form with 12 items across three bipolar dimensions (good-bad mood, awake-tired, calm-nervous) using a five-point Likert scale ranging from ‘1 = not at all’ to ‘5 = very’ (Steyer et al., 2004). The summary score for each dimension ranges from 4 to 20, with higher scores indicating a higher current well-being. The MDBF demonstrates good psychometric properties with an internal consistency of Cronbach's *α* = 0.730 to 0.880 (Steyer et al., 2004).

#### 2.5.5 Emotion regulation

As a secondary outcome and within the EMA, a translated German version of the short form of the Difficulties in Emotion Regulation Scale (DERS-SF) assesses emotion regulation with 18 items on a five-point Likert scale ranging from ‘1 = almost never’ to ‘5 = almost always’ (Kaufman et al., 2016). The summary score ranges from 18 to 90, with higher scores indicating greater difficulties in emotion regulation. The DERS-SF demonstrates good psychometric properties with internal consistency ranging from Cronbach's *α* = 0.890 to 0.910 (Kaufman et al., 2016).

#### 2.5.6 Stress

As a further secondary outcome, the German version of the Perceived Stress Scale 4 (PSS-4) assesses perceived stress with four items on a five-point Likert scale ranging from ‘0 = never’ to ‘4 = very often’ (Klein et al., 2016). The summary score ranges from 0 to 16, with higher scores indicating greater stress. The PSS-4 demonstrates good psychometric properties with Cronbach's *α* = 0.840 (Klein et al., 2016).

#### 2.5.7 Usability and acceptance

The last secondary outcome is the German version of the Mobile App Rating Scale (MARS-G), which captures the quality of mHealth applications on five dimensions (engagement, functionality, aesthetics, subjective quality and information quality) with 29 items, rated on various five-point scales (Messner et al., 2020). The average score ranges from 1 to 5, with higher scores indicating greater app quality. The MARS-G demonstrates acceptable to good psychometric properties, with omega coefficients ranging from *ω* = 0.740 to 0.910 (Messner et al., 2020). Additionally, 38 self-generated items specifically tailored to the content of the *Mental Health Guide* were administered to capture usability and acceptance, e.g., usage of the *app* was assessed using the following item: ‘how often did you use the *Mental Health Guide*?’ on a five-point scale ranging from ‘1 = several times a day’ to ‘6 = never’. A detailed description of all self-generated items can be found in the appendix (Table B).

### 2.6 Analysing strategy

For all statistical analysis and other calculations, the open source software RStudio (R version 4.2.0 (2022-04-22)) is used. Participants with incomplete data sets (‘NA’) are removed before the calculations to ensure the same number of data points for each person. Participants from the IG, who reported that they have never used the app, are also removed before calculations. The significance level for all analyses is α = 0.05. To evaluate the impact of the intervention on MHL as well as secondary outcomes, mixed-measures analyses of variance (ANOVA) are conducted with time (T0, T1, T2, T3) as the within-subject factor and group (IG vs. CG) as the between-subject factor. Additionally, multilevel models with random intercepts and polynomial time variables are estimated to evaluate potential non-linear patterns. Adding age and gender as covariates led to model convergence issues, indicating that the available data may not support the estimation of more complex models. Given the high drop-out rates, the same analyses were repeated for the intervals T0–T1 and T1–T2, with Bonferroni correction applied. These served to provide a more differentiated view of the effects and mitigate the impact of missing data.

In exploratory analyses, the treatment effect is further investigated: The moderating effect of app usage and acceptance is analysed for significant outcomes. To evaluate the predictive capability of everyday mood dynamics and emotion regulation capacities on the outcome measures, the EMA data are analysed using time-series analyses. Autoregressive integrating moving average (ARIMA) modelling is applied to capture the temporal structure of individual-level EMA variables, including both indicators of well-being (MDBF) and emotion regulation (DERS-SF). Based on these models, 7-day forecasts are generated to examine predictive patterns over time. In addition to model-based forecasting, the Mean Squared Successive Differences are calculated for each EMA variable as an indicator of intraindividual variability. This metric quantifies short-term fluctuations in emotional states and regulatory capacities and is commonly used in EMA research to assess affective instability and psychological flexibility (Seizer et al., 2022, Seizer et al., 2024).

Anonymised data and full reproducible code are available on OSF (https://osf.io/4wh5x/?view_only=040375cd3a56472597e788b97e5b8f28).

## 3 Results

### 3.1 Mental health literacy

**Fig. 2:**
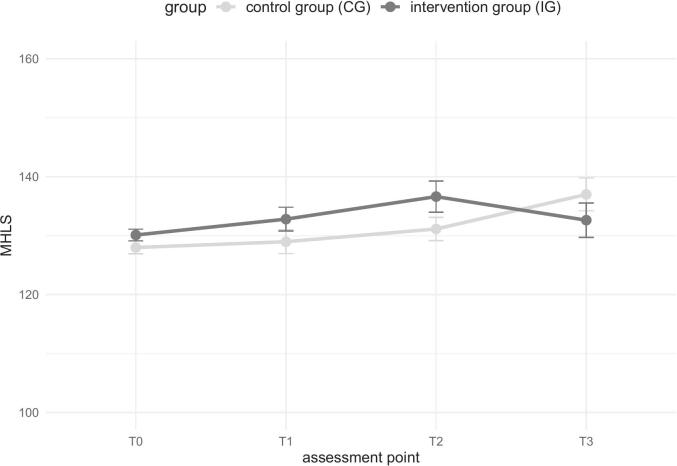
MHL over time per group.

Overall, participants show high MHL in the MHLS at the beginning of the study, with a small increase over time in both groups (see Table 4 and Fig. 2). A significant interaction effect (*F*(1, 72) = 4.10, *p* = .047, *d* = 0.20) has been found for T0 to T1 when the assessment points are considered individually. The IG shows a greater improvement than the CG. No significant interaction effect is observed for T1 to T2. Considering all assessment points from T0 – T2, no significant interaction effect has been shown, *F*(2, 108) = 2.44, *p* = .092, *d* = 0.18, nor a main effect of group, *F*(1, 54) = 0.21, *p* = .650, *d* = 0.11. A significant main effect of time has been found, *F*(2, 108) = 9.35, *p* < .001, *d* = 0.36. Both groups show increased MHL over time. The additionally calculated multilevel models show the same result pattern. For T0 to T2, a significant main effect of time has occurred, *B* = 1.06, *SE* = 0.47, *t*(134) = 2.23, *p* = .027. All further statistical parameters are listed in the Appendix (Tables D–F).

**Table 4:** Descriptive characteristics for all outcome measures over time.

		IG		CG	
		*M*	*SD*	*M*	*SD*
MHLS	T0	130.12	11.47	128.00	12.24
	T1	132.12	10.36	128.98	13.92
	T2	136.63	11.59	131.14	12.81
	T3	132.63	12.64	137.00	11.13
SDQ – Total difficulties	T0	13.80	5.45	13.30	5.44
	T1	11.46	5.64	13.50	5.66
	T2	11.77	4.42	13.36	6.06
	T3	13.89	6.57	14.75	7.59
SDQ – Prosocial behaviour	T0	8.78	1.65	8.70	1.44
	T1	8.88	1.51	8.63	1.50
	T2	8.70	1.47	8.90	1.45
	T3	8.58	1.77	9.25	1.53
WHO-5	T0	18.55	4.93	18.37	5.09
	T1	16.75	5.39	16.89	5.59
	T2	15.77	4.77	18.45	4.85
	T3	17.74	6.01	17.38	5.70
DERS-SF	T0	41.82	9.23	42.59	11.57
	T1	38.08	9.31	40.00	10.80
	T2	37.47	9.16	39.76	11.57
	T3	39.79	11.58	42.69	12.79
PSS-4	T0	11.17	3.14	11.08	3.47
	T1	10.54	3.44	10.43	3.71
	T2	9.50	3.83	10.67	3.57
	T3	11.00	3.28	11.12	3.54

Note. T0 with *n* = 158 in the IG and *n* = 164 CG, T1 with *n* = 37 in the IG and *n* = 57 CG, T2 with *n* = 38 in the IG and *n* = 45 CG, T0 with *n* = 21 in the IG and *n* = 18 CG.

### 3.2 Difficulties and prosocial behaviour

On average, participants from both groups show normal difficulties in the SDQ at T0 (see Table 4 and Appendix Fig. A). Considering only T0 to T1, a significant interaction effect has been found, *F*(1, 72) = 5.809, *p* = .018, *d* = 0.20. The IG shows a greater decrease in difficulties than the CG. From T1 to T2 no significant interaction effect is observed. Including all assessment points, no significant interaction effect has been detected, *F*(2, 106) = 1.71, *p* = .186, *d* = 0.13. There are no significant main effects of time (*F*(2, 106) = 0.05, *p* = .955, *d* = 0.02) nor group (*F*(1, 53) = 0.41, *p* = .710, *d* = 0.09). The additionally calculated multilevel models show the same result pattern. For T0 to T1, a significant interaction between time and group has occurred, *B* = −2.03, *SE* = 0.77, *t*(72) = −2.65, *p* = .010.

IG and CG also show normal prosocial behaviour in the SDQ at T0 and remain stable over almost all assessment points (see Table 4). From T0 to T1 no significant interaction effect is observed (*F*(1, 53) = 1.64, *p* = .205, *d* = 0.00). Considering T1 to T2, a significant interaction effect has been observed, *F*(1, 53) = 7.50, *p* = .008, *d* = 0.23. Considering all assessment points from T0 to T2, a significant interaction effect has been found, *F*(2, 106) = 3.33, *p* = .040, *d* = 0.22. The same pattern is found considering multilevel models (see Appendix, Tables E–F), with a significant interaction between time and group for T1 to T2, *B* = −0.76, *SE* = 0.29, *t*(53) = −2.59, *p* = .012.

### 3.3 Well-being

**Fig. 3:**
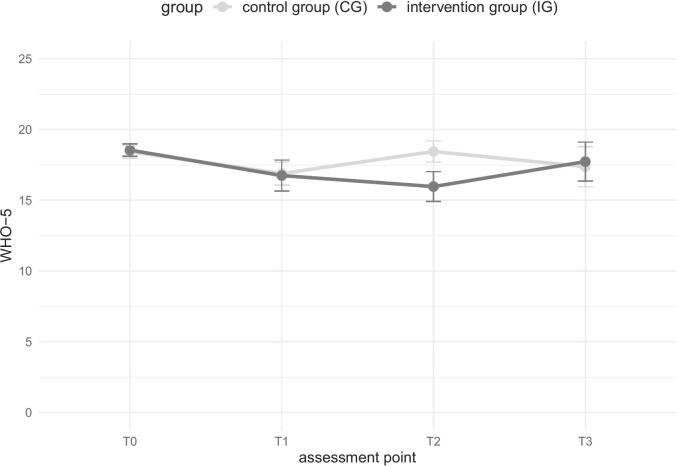
Well-being over time per group.

Overall, participants show higher levels of well-being in the WHO-5 at T0, with small decreases over time in both groups (see Table 4 and Fig. 3). A significant main effect of time (*F*(1, 69) = 5.18, *p* = .026, *d* = 0.00) has been found for T0 to T1 when the assessment points are considered individually. Both groups show a decrease in well-being. No significant interaction effect (*F*(1, 69) = 0.13, *p* = .723, *d* = 0.00) nor a main effect of group has been found (*F*(1, 69) = 0.01, *p* = .936, *d* = 0.00). From T1 to T2 no significant interaction effect is observed (see Appendix Table E). Considering all assessment points T0-T2, no significant interaction effect has occurred, *F*(2, 106) = 1.66, *p* = .196, *d* = 0.22, as well as no significant main effect of time (*F*(2, 106) = 1.88, *p* = .158, *d* = 0.24), nor group (*F*(1, 53) = 0.12, *p* = .729, *d* = 0.06).

Regarding the multilevel models, a significant interaction effect has been found from T0 to T2, *B* = −1.26, *SE* = 0.64, *t*(129) = −1.98, *p* = .050. Consistent with the results from T0 to T1, a significant main effect of time has occurred, *B* = −1.42, *SE* = 0.70, *t*(69) = −2.04, *p* = .045. Furthermore, a significant effect of time has also been found for T1 to T2, *B* = 1.89, *SE* = 0.77, *t*(53) = 2.45, *p* = .017.

### 3.4 Emotion regulation

Participants show mediocre emotion regulation capacities on the DERS-SF in both groups at T0, with a slight improvement over time (see Table 4 and Appendix Fig. C). Considering only the assessment points T0 and T1, a significant main effect of time has been found, *F*(1, 69) = 16.04, *p* < .001, *d* = 0.02, but no interaction effect (*F*(1, 69) = 3.94, *p* = .051, *d* = 0.20) nor a main effect of group (*F*(1, 69) = 0.03, *p* = .869, *d* = 0.00). Both groups show a decrease in emotion regulation difficulties. From T1 to T2, no significant interaction effect is observed (see Appendix Table D). Considering all assessment points T0-T2, no significant interaction effect has occurred, *F*(2, 106) = 1.08, *p* = .343, *d* = 0.09, nor a main effect of group, *F*(1, 53) = 0.12, *p* = .730, *d* = 0.09. There is a significant main effect of time, *F*(2, 106) = 5.92, *p* = .004, *d* = 0.23.

Contrary to these findings, the multilevel models for T0 to T2 have shown a significant interaction effect, *B* = −1.62, *SE* = 0.80, *t*(129) = −2.02, *p* = .045. Consistent with these findings, the multilevel models for T0 to T1 have shown a significant main effect of time, *B* = −1.73, *SE* = 0.86, *t*(69) = −2.02, *p* = .048 (see Appendix, Tables E–G).

### 3.5 Stress

Participants show increased stress in the PSS-4 in both groups at T0 with a slight decrease over time (see Table 4 and Appendix, Fig. D). No significant interaction effects have been found when considering the individual assessment points T0-T1 (*F*(1, 69) = 0.85, *p* = .849, *d* = 0.00) and T1-T2 (*F*(1, 54) = 0.00, *p* = .968, *d* = 0.00). Considering all assessment points from T0 to T2, no significant interaction effect has occurred, *F*(2, 106) = 0.05, *p* = .953, *d* = 0.03, as well as no main effect of time (*F*(2,106) = 1.11, *p* = .332, *d* = 0.16) nor group (*F*(1, 53) = 0.02, *p* = .896, *d* = 0.03). Consistent with these findings, the same results have occurred within the multilevel models. All further statistical parameters can be found in the Appendix (Tables C–F).

### 3.6 Exploratory analyses

#### 3.6.1 Moderating effect of app usage and acceptance

On average, participants rate the quality of the app relatively high (*M* = 3.91, *SD* = 1.19). A detailed description of the rating dimensions is provided in Table 5. 55.13 % have used the app once a week or more often, 41.02 % have used the app every few weeks, and 3.85 % have never used the app. A significant moderating effect of acceptance has been found for overall well-being, as a significant interaction between time and app acceptance has occurred, *B* = 3.18, *SE* = 1.41, *t*(69) = 2.26, *p* = .027. Higher app acceptance strengthens the positive effect of time on overall well-being. No further moderating effects of acceptance or app usage have occurred (see Appendix, Tables H and I).

**Table 5:** Ratings of the app on MARS-G.

	*M*	*SD*
Engagement	3.71	0.60
Functionality	3.95	1.31
Aesthetics	3.77	1.62
Information	4.20	1.71
App quality	3.91	1.19
App subjective quality	2.84	0.59
Awareness	4.26	1.69

#### 3.6.2 Everyday mood dynamics and emotion regulation

During EMA, both groups show mediocre emotion regulation capacities on average (*M*_IG_ = 39.67, *SD*_*IG*_ = 5.65, *M*_CG_ = 40.26, *SD*_*CG*_ = 6.59). The performed time series analyses have shown significant autoregressive dependencies at lag 1 for the IG (φ_1_ = 0.86, *t*(847) = 34.37, *p* < .001) and the CG (φ_1_ = 0.87, *t*(824) = 41.92, *p* < .001). The figures depicting the individual time courses of participants can be found in the Appendix (Figs. E and F).

As for everyday mood dynamics, the current well-being shows similar patterns. IG and CG show on average good mood (*M*_IG_ = 14.84, *SD*_*IG*_ = 3.25, *M*_CG_ = 14.41, *SD*_*CG*_ = 3.45), more calmness (*M*_IG_ = 13.76, *SD*_*IG*_ = 3.32, *M*_CG_ = 13.20, *SD*_*CG*_ = 3.51) and awakeness (*M*_IG_ = 11.38, *SD*_*IG*_ = 3.66, *M*_CG_ = 11.29, *SD*_*CG*_ = 3.63). The time series analyses have shown significant autoregressive dependencies at lag 1 for the three dimensions of current well-being in both groups (see Table 6). The figures depicting the individual time courses of participants can be found in the Appendix (Figs. G and H).

**Table 6:** results from the autoregressive models for current well-being.

	φ_1_	*t*	*p*
*IG*			
Good-bad mood	0.79	34.37	<.001
Calm-nervous	0.82	19.44	<.001
Awake-tired	0.79	72.55	<.001

*CG*			
Good-bad mood	0.79	21.46	<.001
Calm-nervous	0.75	15.64	<.001
Awake-tired	0.75	14.16	<.001

Note. Degrees of freedom for IG *n* = 847, for CG *n* = 824.

## 4 Discussion

The aim of this study was to improve mental health and increase MHL of young adults. Within a randomised controlled study design, this study has evaluated the effectiveness of a mHealth app, the *Mental Health Guide*, as a universal prevention for young adults. As expected, participants using the app (IG) improved their MHL compared to the CG in the post-assessment, as well as in the 3-month follow-up. Furthermore, we hypothesised that the app would improve different aspects of participants' mental health. Different results have been obtained for the various aspects of mental health: Contrary to our expectations, the overall well-being of participants from both groups deteriorated between pre- and post-assessment. App acceptance moderated the effect of time on well-being, with higher acceptance rates leading to a better well-being over time. However, emotion regulation capacities from both groups improved. In line with our assumptions, participants from the IG showed less problematic behaviour in the post-assessment compared to the CG, as well as more prosocial behaviour over the time course from post-assessment to the first follow-up period. The app showed no effect on participants' stress.

Overall, these results offer initial support for the short-term effectiveness of mHealth interventions that may contribute to universal prevention efforts. These findings are in line with previous research (Hetrick et al., 2016; Loechner et al., 2018), where effect sizes have been rather small and evidence for long-term effectiveness has not yet been demonstrated. This is partly due to the nature of the study being a universal prevention measure (Hetrick et al., 2016), but also due to the characteristics of the sample size, which impair statistical power. Over the course of the study, there has been a high drop-out rate up to 80 % after nine months from the baseline assessment (T3), which is in line with previous research of real-world mHealth interventions (Fleming et al., 2018). Self-guided interventions are generally characterised by lower adherence, which is mirrored in lower overall effectiveness (Firth et al., 2017; Wright et al., 2019). Due to the high dropout rate and the apparent non-random nature of the missingness, no data imputation was conducted, which is an important limitation of the present study. This approach, while methodologically cautious, may have introduced bias and limits the generalizability of the findings. Future research should place greater emphasis on minimizing attrition and consider the application of intention-to-treat analyses to account for missing data more robustly. Nevertheless, regardless of studies that use additional gratification, our study provides a good representation of the use of mHealth interventions under real-world conditions: In over 10,000 digital mental health apps, only 11 peer-reviewed publications have analysed uptake and usage data in such real-life settings. The completion rate was between 44 and 99 % in RCTs but dropped to 1–28 % when looking at real-world usage (Fleming et al., 2018).

The slightly higher dropout rate in the IG may be explained by their immediate access to the app, which also served as reimbursement. In contrast to the CG, who received access only after completing the follow-up assessment, participants from the IG may have had less incentive to complete all assessment points. A similar trend of higher drop-out rates in intervention groups has also been reported in other online intervention studies (Andersson, 2002; Bewick et al., 2010; Geraghty et al., 2010), with group membership being a significant predictor of adherence and consequently study drop-out (Beatty and Binnion, 2016). This may be attributable to the increased workload associated with intervention participation as well as the potentially distressing or uncomfortable nature of engaging with personal or sensitive topics during the intervention.

Further characteristics associated with smaller effect sizes include a rather selective sample. Our sample has shown high MHL from the beginning of the study and consists mainly of female participants in higher education settings. Due to the combination of these rather protective factors for mental health (Siepmann, 2012) the achievement of larger intervention effects is impeded. Nevertheless, indications of an indirect effect on well-being have been found. In line with previous research (Schiller et al., 2014), participants' engagement with the app's content led to an expansion of their MHL and increased well-being. For further behavioural changes, the distribution of more practical implementations next to the provision of information would have been necessary. To generalise the effects found, future studies might want to include more heterogeneous samples.

On the other hand, the age distribution within the sample demonstrates greater heterogeneity than initially anticipated, extending into late adulthood (see Appendix Table A). Despite this variability, over half of the participants can be classified as young adults (Arnett et al., 2014), with a comparable age group distribution observed at post- and follow-up time points (see Appendix A). This age focus is likely attributable to the open inclusion criteria and a recruitment strategy primarily designed to reach younger populations, for example, through social media platforms. However, such broad recruitment approaches inherently reduce control over the demographic characteristics of the sample. Consequently, the findings cannot be generalised exclusively to young adults. Given the high prevalence of mental health disorders (Solmi et al., 2022), early preventive interventions are particularly crucial in younger populations. Nonetheless, as indicated by our data, there appears to be interest in prevention efforts across a broader age spectrum, suggesting the relevance of such interventions beyond youth-focused groups.

Besides the mentioned limitations above, the chosen study design also has the strength of evaluating the use of mHealth interventions as closely as possible under real-world conditions that participants experience. Without many instructions, the majority of participants have shown regular use of the app. Participants' evaluations are consistent with the results of earlier studies, in which mHealth programmes can be seen as additional resources in prevention efforts (Beames et al., 2023). Considering the high individual fluctuations of current well-being and emotion regulation (Seizer et al., 2024), the use of mHealth programmes offers the opportunity to combine technical advantages, e.g., temporal and local flexibility and independence (Robinson et al., 2016; Paganini et al., 2018), with participants' individual needs. Therefore, the study results contribute important conclusions for future universal prevention: This initial use of mHealth applications represents a promising approach to introducing young people to mental health education. As previous research suggests, a consequential effect of strengthening MHL is reduced stigmatisation and increased likelihood of seeking help (Henderson et al., 2013; Gorczynski et al., 2017; Lui et al., 2024). To achieve long-term effects, interventions should emphasize regular prompts and the practical applicability of information in daily life. A more user-centered and participatory approach (Vogel et al., 2024), can enhance an individualised user experience and potentially reduce drop-out rates.

## 5 Conclusion

As a first-of-its-kind study, this study evaluated the use of a mHealth universal prevention measure for young adults. The results offer a valuable foundation, suggesting that low-threshold, educative interventions may contribute to improving well-being in young adults. The approach of combining a randomised controlled study design with EMA helped to investigate participants' changes in a naturalistic setting in their everyday life. In order to generalise the effects found, future studies are needed that take into account the challenges addressed.

## CRediT authorship contribution statement

JL, CG, and IB conceptualized the study and its design, OK was mainly responsible for managing the study, recruitment, and conducting the randomised controlled trial with the support and supervision of IB and JL. OK wrote the first draft of this manuscript and was responsible for the statistical analyses. IB, JL, and CG reviewed the final version and commented on all parts of the manuscript.

## Declaration of Generative AI and AI-assisted technologies in the writing process

During the preparation of this work the authors used ChatGPT in order to improve language. After using this tool/service, the authors reviewed and edited the content as needed and take full responsibility for the content of the publication.

## Funding

This study is financed by own funds (appointment funds Prof. Löchner).

## Declaration of competing interest

None.

## Appendix A Supplementary data

Supplementary material Image 1
