# Supplementary material for: Prevention of mental health issues in the young: A randomised controlled evaluation of an e-mental health application for young adults to enhance mental health literacy
Source: Internet Interv. 2025 Sep 17;42:100874. doi: 10.1016/j.invent.2025.100874 (PMC12482343; doi:10.1016/j.invent.2025.100874)
Supplement: Supplementary file 1 — Supplementary material [file mmc1.docx]

**Appendix. Supplementary analyses data**

**Table A (relative) frequency of age ranges for T0 to T2**

|  | T0 | T1 | T2 |
| --- | --- | --- | --- |
| 14-18 years | 16.8% (n = 54) | 5.3% (n = 5) | 9.6% (n = 8) |
| 18-25 years | 42.7% (n = 137) | 45.7% (n = 43) | 42.2% (n = 35) |
| 25-35 years | 25.9% (n = 83) | 33.0% (n = 31) | 37.4% (n = 31) |
| 35-45 years | 8.4% (n = 27) | 8.5% (n = 8) | 6.0% (n = 5) |
| 45-59 years | 6.1% (n = 20) | 7.5% (n = 7) | 5.0% (n = 4) |

**Table B self-generated items app acceptance and usability**

| Item | Scale |
| --- | --- |
| Have you used (m)Health apps before? | Yes \| No |
| How many (m)Health apps did you use? | Open format |
| How often did you use (m)Health apps? | Several times a day \| once a day \| several times a week \| once a week \| every few weeks |
| How useful do you find (m)Health apps? | very useful \| useful \| less useful \| not useful at all |
| How often did you use the Mental Health Guide? | several times a day \| once a day \| several times a week \| once a week \| every few weeks \| not at all |
| How easy did you find the individual modules of the Mental Health Guide to understand? | very comprehensible \| comprehensible \| partially comprehensible \| less comprehensible \| not comprehensible at all |
| How helpful did you find Module e.g., #1 Body?* | very helpful \| helpful \| not very helpful \| not helpful at all |
| How entertaining did you find Module e.g., #1 Body?* | very entertaining \| entertaining \| not very entertaining \| not entertaining at all |
| Have you learnt anything new from Module e.g., #1 Body?* | Learnt a lot \| learnt something \| learnt few \| learnt nothing |

*****item provided separately for each module

**Table C ANOVA results for T0 to T1**

|  | *F* | *df* | *p* | *d* |
| --- | --- | --- | --- | --- |
| **MHL (MHLS)** |  |  |  |  |
| Group | 0.48 | 1, 72 | 0.492 | 0.20 |
| Time | 0.15 | 1, 72 | 0.701 | 0.00 |
| Time x group | 4.10 | 1, 72 | **0.047** | 0.20 |
| **Total Difficulties (SDQ)** |  |  |  |  |
| Group | 0.64 | 1, 72 | 0.425 | 0.20 |
| Time | 0.73 | 1, 72 | 0.397 | 0.00 |
| Time x Group | 5.81 | 1, 72 | **0.018** | 0.20 |
| **Prosocial behaviour (SDQ)** |  |  |  |  |
| Group | 0.06 | 1, 72 | 0.807 | 0.00 |
| Time | 0.15 | 1, 72 | 0.705 | 0.00 |
| Time x Group | 1.64 | 1, 72 | 0.205 | 0.00 |
| **Well-being (WHO-5)** |  |  |  |  |
| Group | 0.01 | 1, 69 | 0.936 | 0.00 |
| Time | 5.18 | 1, 69 | **0.026** | 0.00 |
| Time x Group | 0.13 | 1, 69 | 0.723 | 0.00 |
| **Emotion Regulation (DERS-SF)** |  |  |  |  |
| Group | 0.03 | 1, 69 | 0.869 | 0.00 |
| Time | 16.04 | 1, 69 | **0.000** | 0.26 |
| Time x group | 3.94 | 1, 69 | 0.051 | 0.20 |
| **Stress (PSS-4)** |  |  |  |  |
| Group | 0.00 | 1, 69 | 0.974 | 0.00 |
| Time | 1.57 | 1, 69 | 0.215 | 0.00 |
| Time x Group | 0.85 | 1, 69 | 0.849 | 0.00 |

**Table D ANOVA results for T1 to T2**

|  | *F* | *df* | *p* | *d* |
| --- | --- | --- | --- | --- |
| **MHL (MHLS)** |  |  |  |  |
| Group | 2.86 | 1, 54 | 0.097 | 0.46 |
| Time | 0.54 | 1, 54 | 0.467 | 0.00 |
| Time x group | 0.17 | 1, 54 | 0.681 | 0.00 |
| **Total Difficulties**  **(SDQ)** | |  |  |  |
| Group | 0.46 | 1, 53 | 0.500 | 0.20 |
| Time | 0.10 | 1, 53 | 0.754 | 0.00 |
| Time x Group | 0.77 | 1, 53 | 0.384 | 0.00 |
| **Prosocial behaviour**  **(SDQ)** |  |  |  |  |
| Group | 0.45 | 1, 53 | 0.504 | 0.20 |
| Time | 1.36 | 1, 53 | 0.249 | 0.00 |
| Time x Group | 7.50 | 1, 53 | **0.008** | 0.26 |
| **Well-being (WHO-5)** |  |  |  |  |
| Group | 0.47 | 1, 53 | 0.497 | 0.20 |
| Time | 2.07 | 1, 53 | 0.156 | 0.20 |
| Time x Group | 2.72 | 1, 53 | 0.105 | 0.20 |
| **Emotion Regulation (DERS-SF)** |  |  |  |  |
| Group | 0.01 | 1, 53 | 0.937 | 0.00 |
| Time | 0.19 | 1, 53 | 0.664 | 0.00 |
| Time x group | 0.03 | 1, 53 | 0.856 | 0.00 |
| **Stress (PSS-4)** |  |  |  |  |
| Group | 0.05 | 1, 53 | 0.827 | 0.00 |
| Time | 0.13 | 1, 53 | 0.724 | 0.00 |
| Time x Group | 0.00 | 1, 53 | 0.968 | 0.00 |

**Table E multilevel model results for T0 to T2**

|  | *B* | *SE* | *t* | | | *p* | | | CI | |
| --- | --- | --- | --- | --- | --- | --- | --- | --- | --- | --- |
|  |  |  |  | | |  | | | LB | UB |
| **MHL (MHLS)** |  |  |  | | |  | | |  |  |
| Group | 0.39 | 0.92 | 0.43 | | | 0.670 | | | -1.43 | 2.22 |
| Time | 1.06 | 0.47 | 2.23 | | | **0.027** | | | 0.12 | 1.99 |
| Time x group | 1.30 | 0.83 | 1.57 | | | 0.118 | | | -0.33 | 2.94 |
| **Total Difficulties**  **(SDQ)** |  |  | |  |  | | |  |  |  |
| Group | 0.34 | 0.67 | 0.51 | | | 0.613 | | | -0.98 | 1.66 |
| Time | 0.27 | 0.24 | 1.13 | | | 0.259 | | | -0.20 | 0.74 |
| Time x group | -0.77 | 0.42 | -1.83 | | | 0.070 | | | -1.61 | 0.06 |
| **Prosocial behaviour**  **(SDQ)** | |  |  | | | |  |  | |  |
| Group | 0.13 | 0.19 | 0.69 | | | 0.493 | | | -0.25 | 0.51 |
| Time | 0.07 | 0.08 | 0.82 | | | 0.416 | | | -0.10 | 0.23 |
| Time x group | -0.21 | 0.15 | -1.41 | | | 0.160 | | | -0.49 | 0.08 |
| **Well-being**  **(WHO-5)** |  |  |  | | | |  |  | |  |
| Group | 0.26 | 0.64 | 0.41 | | | 0.680 | | | -1.00 | 1.53 |
| Time | 0.02 | 0.36 | 0.06 | | | 0.953 | | | -0.70 | 0.74 |
| Time x group | -1.26 | 0.64 | -1.98 | | | **0.050** | | | -2.52 | 0.00 |
| **Emotion regulation**  **(DERS-SF)** | |  |  | | | |  |  | |  |
| Group | -1.02 | 1.30 | -0.78 | | | 0.437 | | | -3.59 | 1.56 |
| Time | -0.76 | 0.45 | -1.70 | | | 0.091 | | | -1.65 | 0.12 |
| Time x group | -1.62 | 0.80 | -2.02 | | | **0.045** | | | -3.21 | -0.03 |
| **Stress (PSS-4)** |  |  |  | | |  | | |  |  |
| Group | 0.11 | 0.43 | 0.26 | | | 0.794 | | | -0.73 | 0.96 |
| Time | -0.22 | 0.23 | -0.94 | | | 0.349 | | | -0.68 | 0.24 |
| Time x group | -0.23 | 0.41 | -0.57 | | | 0.569 | | | -1.04 | 0.57 |

*Note.* Degrees of freedom for MHL *n* = 134, for all secondary outcomes *n* = 129

**Table F multi-level model results for T0 to T1**

|  | *B* | *SE* | *t* | *p* | | | CI | |
| --- | --- | --- | --- | --- | --- | --- | --- | --- |
|  |  |  |  |  | | | LB | UB |
| **MHL (MHLS)** |  |  |  |  | | |  |  |
| Group | 2.07 | 1.48 | 1.40 | 0.165 | | | -0.87 | 5.01 |
| Time | -1.62 | 1.12 | -1.44 | 0.153 | | | -3.86 | 0.62 |
| Time x group | 3.50 | 1.88 | 1.86 | 0.068 | | | -0.26 | 7.25 |
| **Total Difficulties**  **(SDQ)** | |  |  | |  |  | |  |
| Group | 0.45 | 0.67 | 0.66 | 0.509 | | | -0.89 | 1.78 |
| Time | 0.54 | 0.46 | 1.19 | 0.239 | | | -0.37 | 1.45 |
| Time x group | -2.03 | 0.77 | -2.65 | **0.010** | | | -3.56 | -0.50 |
| **Prosocial behaviour**  **(SDQ)** |  |  |  | |  |  | |  |
| Group | 0.09 | 0.19 | 0.45 | 0.652 | | | -0.30 | 0.47 |
| Time | -0.11 | 0.16 | -0.69 | 0.489 | | | -0.43 | 0.21 |
| Time x group | 0.31 | 0.26 | 1.16 | 0.251 | | | -0.22 | 0.83 |
| **Well-being (WHO-5)** |  |  |  | |  |  | |  |
| Group | 0.09 | 0.65 | 0.14 | 0.890 | | | -1.21 | 1.39 |
| Time | -1.42 | 0.70 | -2.04 | **0.045** | | | -2.81 | -0.03 |
| Time x group | -0.35 | 1.17 | -0.30 | 0.767 | | | -2.69 | 1.99 |
| **Emotion regulation**  **(DERS-SF)** |  |  |  | |  |  | |  |
| Group | -1.01 | 1.30 | -0.77 | 0.441 | | | -3.60 | 1.59 |
| Time | -1.73 | 0.86 | -2.02 | **0.048** | | | -3.44 | -0.02 |
| Time x group | -2.66 | 1.47 | -1.81 | 0.074 | | | -5.58 | 0.27 |
| **Stress** |  |  |  |  | | |  |  |
| Group | 0.07 | 0.43 | 0.15 | 0.879 | | | -0.79 | 0.93 |
| Time | -0.68 | 0.48 | -1.41 | 0.611 | | | -1.64 | 0.28 |
| Time x group | 0.11 | 0.80 | 0.13 | 0.896 | | | -1.50 | 1.71 |

*Note.* Degrees of freedom for MHL *n* = 72, for all secondary outcomes *n* = 69

**Table G multilevel model results for T1 to T2**

|  | *B* | *SE* | | *t* | *p* | CI | | | |
| --- | --- | --- | --- | --- | --- | --- | --- | --- | --- |
|  |  |  | |  |  | LB | | UB | |
| **MHL (MHLS)** |  |  | |  |  |  | |  | |
| Group | 4.35 | 4.41 | | 0.99 | 0.225 | -4.43 | | 13.13 | |
| Time | 1.59 | 1.35 | | 1.17 | 0.246 | -1.12 | | 1.59 | |
| Time x group | -0.37 | 2.38 | | -0.15 | 0.878 | -5.14 | | 4.41 | |
| **Total Difficulties**  **(SDQ)** | | |  |  |  | |  | |  |
| Group | -3.28 | 1.83 | | -1.79 | 0.077 | -6.93 | | 0.36 | |
| Time | -0.23 | 0.51 | | -0.45 | 0.651 | -1.27 | | 0.80 | |
| Time x group | 0.76 | 0.92 | | 0.83 | 0.412 | -1.09 | | 2.61 | |
| **Prosocial behaviour (SDQ)** |  | |  |  |  | |  | |  |
| Group | 0.94 | 0.52 | | 1.80 | 0.076 | -0.10 | | 1.98 | |
| Time | 0.25 | 0.30 | | 27.7 | 0.130 | -0.08 | | 0.58 | |
| Time x group | -0.76 | 0.29 | | -2.59 | **0.012** | -1.34 | | -0.17 | |
| **Well-being (WHO-5)** |  | |  |  |  | |  | |  |
| Group | 2.00 | 2.28 | | 0.88 | 0.382 | -1.13 | | 1.38 | |
| Time | 1.87 | 0.77 | | 2.45 | **0.017** | -0.73 | | 0.44 | |
| Time x group | -2.33 | 1.38 | | -1.70 | 0.096 | -1.09 | | 0.60 | |
| **Emotion regulation (DERS-SF)** |  | |  |  |  | |  | |  |
| Group | -2.79 | 3.41 | | -0.82 | 0.416 | -9.57 | | 4.00 | |
| Time | 0.40 | 0.89 | | 0.45 | 0.652 | -1.38 | | 2.19 | |
| Time x group | -0.27 | 1.60 | | -0.17 | 0.869 | -3.47 | | 2.94 | |
| **Stress (PSS-4)** |  |  | |  |  |  | |  | |
| Group | 0.25 | 1.54 | | 0.16 | 0.870 | -2.81 | | 3.31 | |
| Time | 0.21 | 0.51 | | 0.42 | 0.675 | -0.81 | | 1.23 | |
| Time x group | -0.41 | 0.91 | | -0.45 | 0.652 | -2.24 | | 1.41 | |

*Note.* Degrees of freedom for MHL *n* = 54, for all secondary outcomes *n* = 53

**Figure A Problematic behaviour over time per group**

**
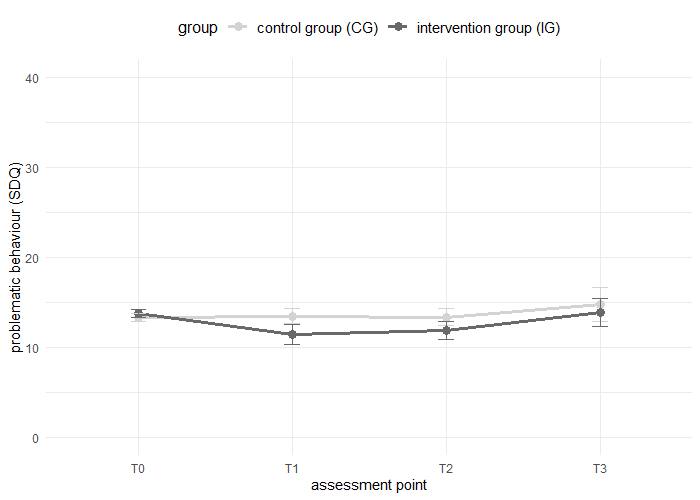
**

**Figure B Prosocial behaviour over time per group**

**
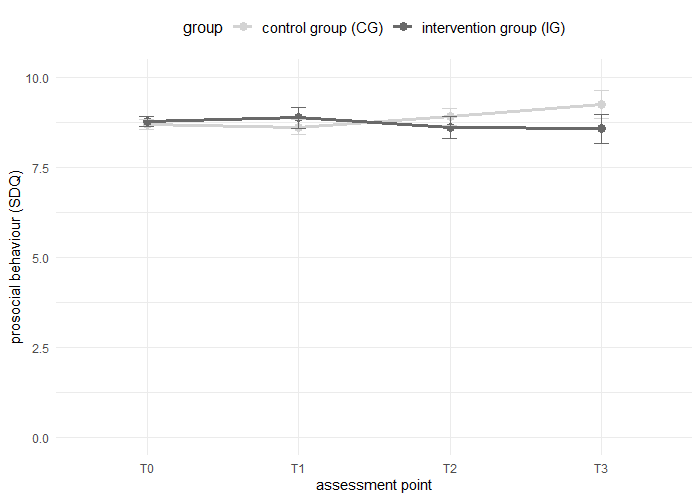
**

**Figure C Emotion regulation over time per group**

**
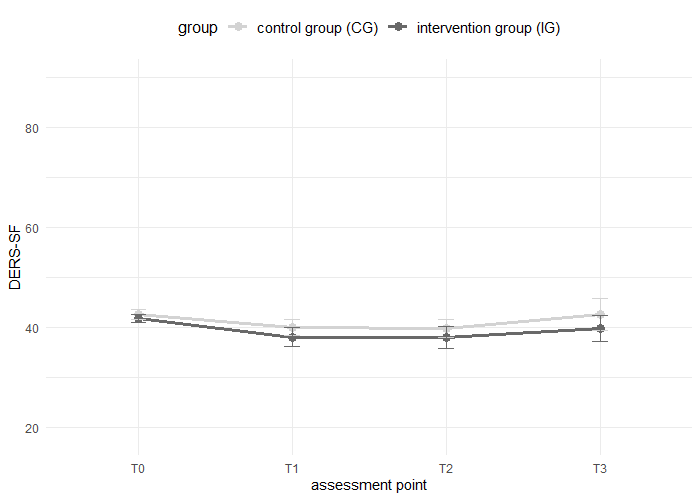
**

**Figure D Stress over time per group**

**
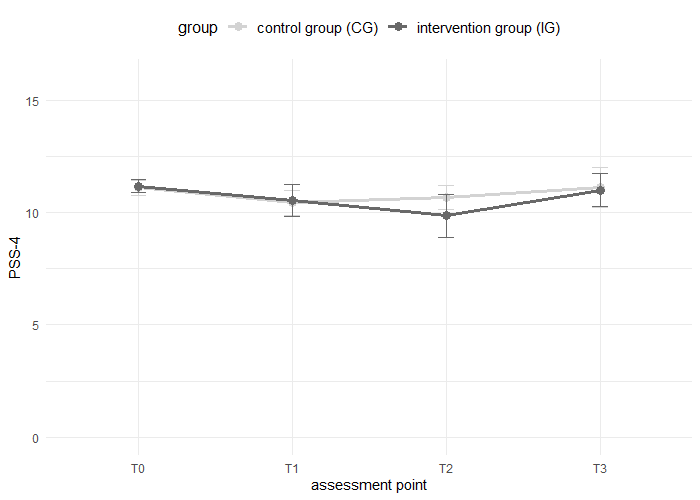
**

**Table H moderating effects of app quality**

|  | *B* | *SE* | *t* | *p* |
| --- | --- | --- | --- | --- |
| MHL (MHLS) | -1.23 | 2.75 | -0.45 | 0.655 |
| Total Difficulties (SDQ) | -1.45 | 1.33 | -1.09 | 0.279 |
| Prosocial Behaviour (SDQ) | -0.63 | 0.40 | -1.55 | 0.125 |
| Well-being (WHO-5) | 3.18 | 1.41 | 2.26 | **0.027** |
| Emotion regulation (DERS-SF) | -2.59 | 2.61 | -0.99 | 0.325 |

*Note.* Degrees of freedom for MHL *n* = 72, for all secondary outcomes *n* = 69

**Table I moderating effects of app usage**

|  | *B* | *SE* | *t* | *p* |
| --- | --- | --- | --- | --- |
| MHL (MHLS) | -0.80 | 1.61 | -0.50 | 0.621 |
| Total Difficulties (SDQ) | 0.94 | 0.73 | 1.28 | 0.205 |
| Prosocial Behaviour (SDQ) | 0.19 | 0.21 | 0.90 | 0.371 |
| Well-being (WHO-5) | -0.62 | 0.72 | -0.86 | 0.392 |
| Emotion regulation (DERS-SF) | -0.61 | 1.38 | -0.44 | 0.663 |

*Note.* Degrees of freedom for MHL *n* = 72, for all secondary outcomes *n* = 69

**Figure E individual time course of emotion regulation in the IG**

**
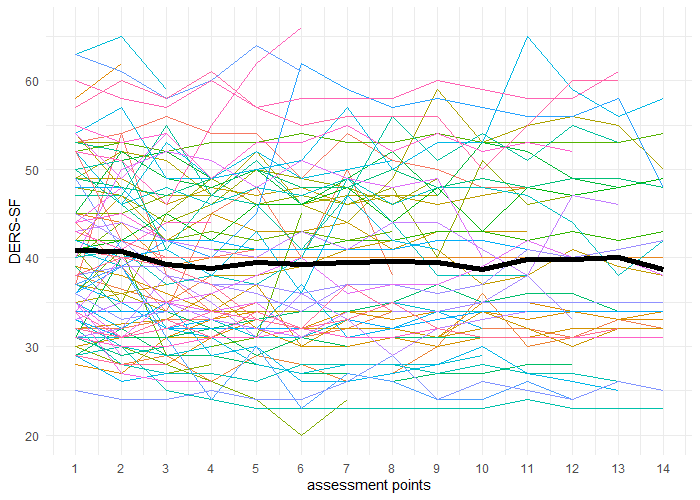
**

**Figure F individual time course of emotion regulation in the CG**

**
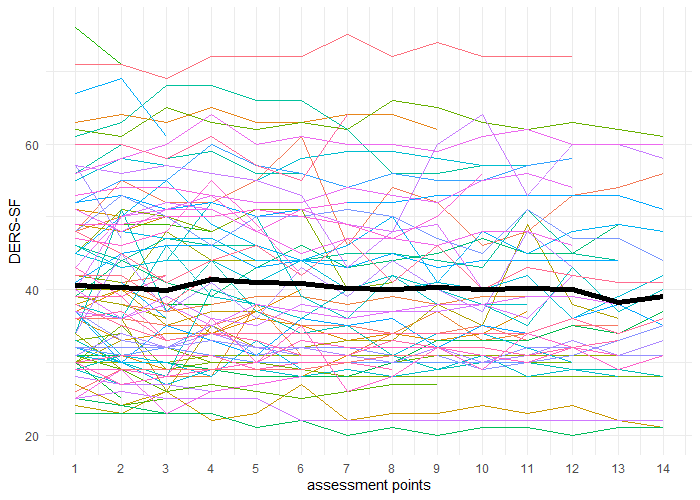
**

**Figure G individual time course of current well-being in the IG**

**
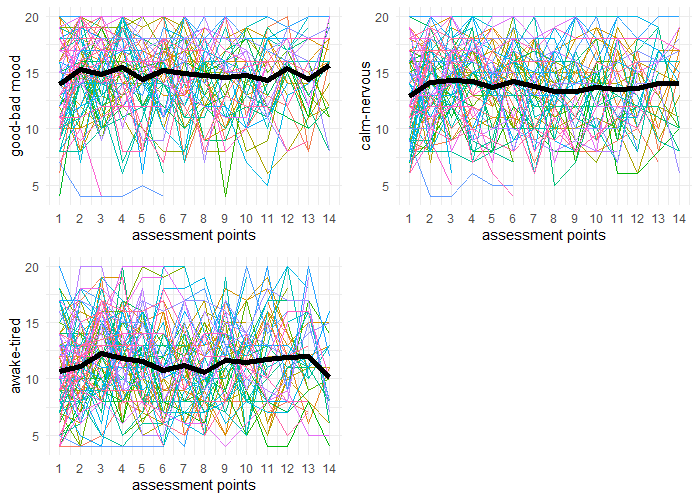
**

**Figure H individual time course of current well-being in the CG**

**
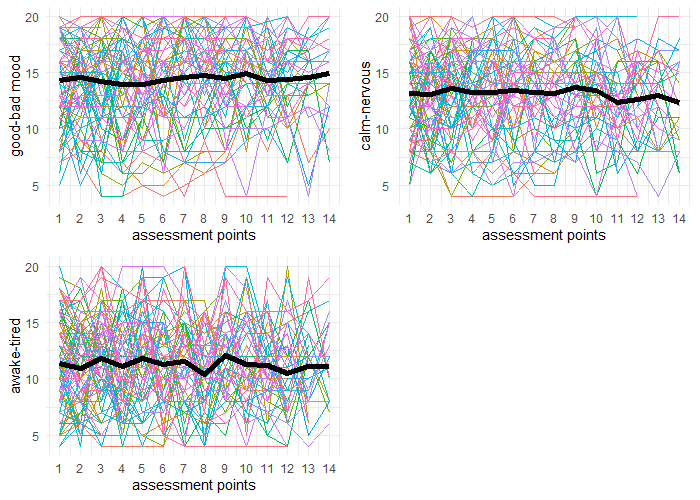
**
